# CNN-XGBoost fusion-based affective state recognition using EEG spectrogram image analysis

**DOI:** 10.1038/s41598-022-18257-x

**Published:** 2022-08-19

**Authors:** Md. Sakib Khan, Nishat Salsabil, Md. Golam Rabiul Alam, M. Ali Akber Dewan, Md. Zia Uddin

**Affiliations:** 1 BRAC University, Dhaka, Bangladesh; 2 Athabasca University, Athabasca, AB Canada; 3 SINTEF Digital, Trondheim, Norway

**Keywords:** Biomedical engineering, Mathematics and computing

## Abstract

Recognizing emotional state of human using brain signal is an active research domain with several open challenges. In this research, we propose a signal spectrogram image based CNN-XGBoost fusion method for recognising three dimensions of emotion, namely arousal (calm or excitement), valence (positive or negative feeling) and dominance (without control or empowered). We used a benchmark dataset called DREAMER where the EEG signals were collected from multiple stimulus along with self-evaluation ratings. In our proposed method, we first calculate the Short-Time Fourier Transform (STFT) of the EEG signals and convert them into RGB images to obtain the spectrograms. Then we use a two dimensional Convolutional Neural Network (CNN) in order to train the model on the spectrogram images and retrieve the features from the trained layer of the CNN using a dense layer of the neural network. We apply Extreme Gradient Boosting (XGBoost) classifier on extracted CNN features to classify the signals into arousal, valence and dominance of human emotion. We compare our results with the feature fusion-based state-of-the-art approaches of emotion recognition. To do this, we applied various feature extraction techniques on the signals which include Fast Fourier Transformation, Discrete Cosine Transformation, Poincare, Power Spectral Density, Hjorth parameters and some statistical features. Additionally, we use Chi-square and Recursive Feature Elimination techniques to select the discriminative features. We form the feature vectors by applying feature level fusion, and apply Support Vector Machine (SVM) and Extreme Gradient Boosting (XGBoost) classifiers on the fused features to classify different emotion levels. The performance study shows that the proposed spectrogram image based CNN-XGBoost fusion method outperforms the feature fusion-based SVM and XGBoost methods. The proposed method obtained the accuracy of 99.712% for arousal, 99.770% for valence and 99.770% for dominance in human emotion detection.

## Introduction

Emotion is fundamentally related to the way people interact with each other as well as with the devices. A human being can infer another human being’s emotional state, through verbal communication, facial expressions^1^, body language, and act in any specific situation. On the other hand, machines have no understanding or features related to emotions. Yet, people are heavily dependent on the technology and the technology has become a part of our daily life. In this respect, emotion recognition, especially through using brain signals, is an active area of research with several open challenges. This area of research is commonly known as Brain Computer Interfacing (BCI).

Up to now some BCI systems make use of brain functions to exchange information and operate devices without requiring neuromuscular brain production pathways^2^. BCI based approaches are being continuously improved through using better feature extraction, band extraction, scaling, and classification models and by using low cost devices such as wearable devices for the day to day activities^3^. Approaches using Electroencephalogram (EEG) signals for emotion identification have problems surrounding the induction of emotional states and the extraction of traits for optimal classification. Approaches based on EEG signals use expensive medical graded equipment, which makes brain signal analysis on daily basis unfeasible^3^. The neural network-based approaches which uses visual and audio inputs typically lacks accuracy, due to consistency deviation of human emotion over voice and expression, and will not bring high-end results. These techniques are not effective for reticent people with physical disabilities when they are unable to articulate their feelings or to interact fully^4^.

**Figure 1:**
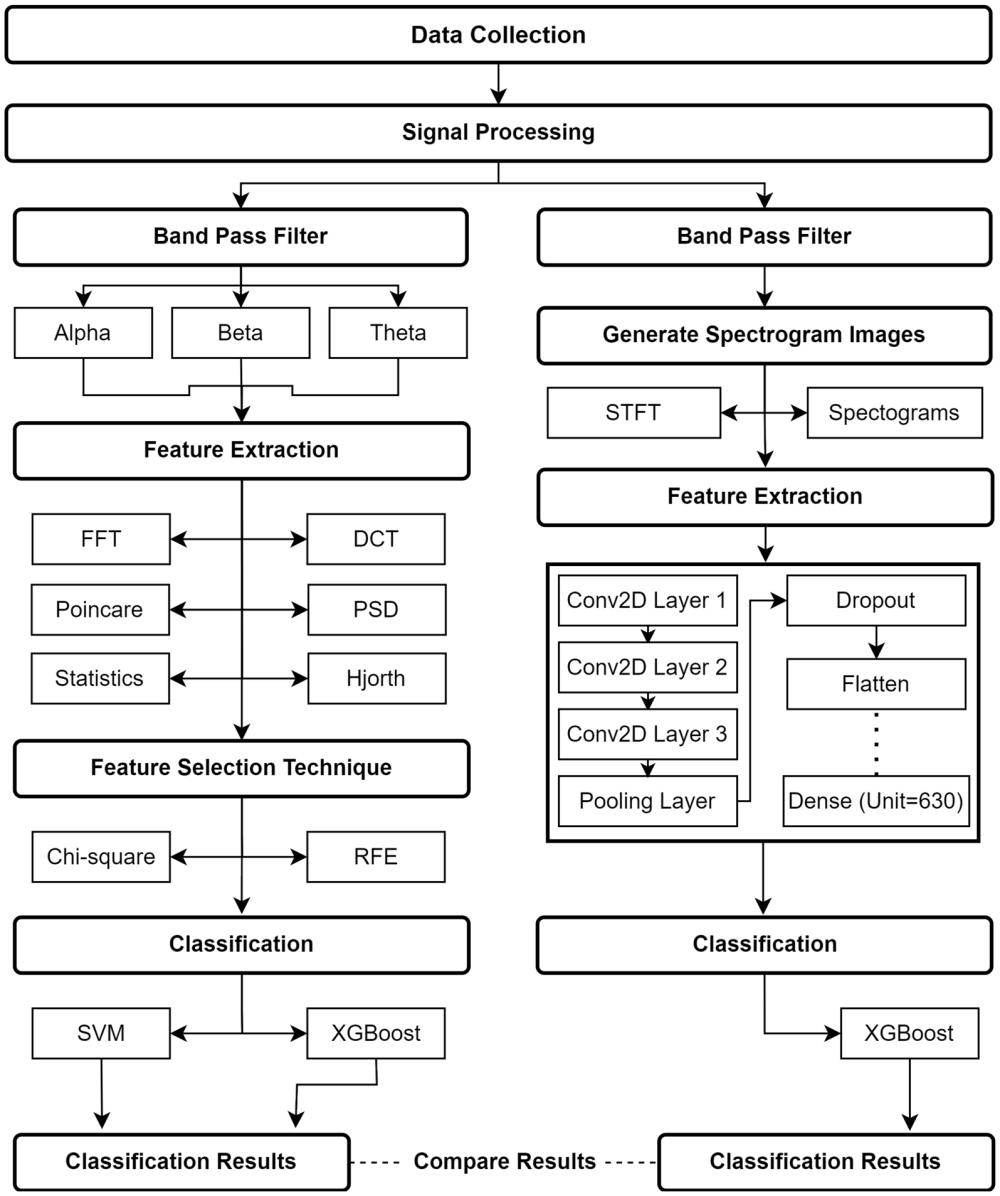
An overview of the proposed method.

In this study, we aim at recognising human emotion through using EEG signals. We focus on recognising three dimensions of emotion. These are arousal (calm or excitement), valence (positive or negative feeling) and dominance (without control or empowered). We used a publicly available DREAMER^3^ dataset consisting of EEG signals tested on multiple stimulus along with self-evaluation ratings. In our proposed spectrogram image-based 2DCNN-XGBoost fusion approach, we calculate the Short-Time Fourier Transform (STFT) of the existing EEG signals from the dataset and convert them into RGB images for further image processing. The key contributions of the proposed method are:

1. Analysis of the RGB spectrogram images of EEG signals: We calculate the STFT of the EEG signals and convert them into RGB spectrogram images in order to extract features using a 2D Convolutional Neural Network (CNN). We use the 2DCNN layers to train the model using the obtained spectrogram images and another dense layer to retrieve the trained features.
2. Extracting more relevant features: Fast Fourier Transformation, Discrete Cosine Transformation, Poincare, Power Spectral Density, Hjorth parameters, and some statistical features are among the feature extraction strategies used in this study for comparison with our suggested method. The Chi-square and Recursive Feature Elimination strategies are also used to select discriminative features. As previously stated, we extract features from the 2DCNN layer using spectrogram images of the EEG signals. This results in more relevant and useful features.
3. Performance study on the DREAMER dataset: We use the DREAMER dataset for testing our proposed method (i.e., Fig. 1) and comparing it against the existing solutions. In our 2DCNN-XGBoost fusion-based approach, we generate the spectrogram images and retrieve the features from the training layer of 2DCNN to use them for further classification using XGBoost. As a result, we achieved a very high accuracy of 99.712% for arousal, 99.770% for valence, and 99.770% for dominance using this method.

“Related works” dives into the related study of different techniques for affect detection and classification models. “Proposed method” describes the proposed method and the procedures used in the current study. “Results and discussion” presents the findings and performance of our proposed method, along with the best approach derived from our method. “Conclusion” finishes the report with an overview of the entire experiment and its prospects. Section 6 provides information on data availability.

## Related works

There are numerous methods for detecting emotions from brain signals. Various approaches were employed to influence data collection, such as facial expressions, peripheral physiological signs, and many more. Furthermore, pattern recognition algorithms for impact recognition involve collecting data on an individual’s emotional state in the brain while expressing an emotion. Emotion has an essential role in interpersonal connection and correspondence between people. Affective computing employs various emotion recognition models and explains numerous hypotheses about how emotions develop. Because of exponential progress in machine learning, deep learning, and countless real-world applications, EEG data processing for emotion identification has enriched in recent years.

Given the growing interest in affective computing and emotional state recognition, Katsigiannis et al.^3^ proposed DREAMER, a database for measuring emotions evoked by audio-visual stimuli. Each participant rated their emotional response on the valence, arousal, and dominance scales after watching each of the 18 video stimuli designed to evoke specific emotions. In addition, participant-specific classification tests for the valence, arousal, and dominance scales were conducted to generate baseline findings for the proposed database in terms of classification accuracy and F1 scores. Random voting and class ratio voting were outperformed by classification based on EEG and ECG-based features, as well as their fusion. They also agreed with an earlier study that employed non-portable medical devices, which can pose additional research challenges.

A new generation of emotion detection model is proposed by Tuncer et al.^5^, to demonstrate a highly accurate emotion recognition system using EEG signals, a deep classifier, and hand-crafted feature construction. Pre-processing, fusion feature synthesis with Led-Pattern and statistical feature generator, discriminative feature selection with RFI, Chi2, and classification with SVM are the four main phases of this model using the GAMEEMO and DREAMER datasets. The outcomes illustrate the efficacy of the proposed emotion recognition model. However, there are several research gaps, such as a lack of more relevant features in the feature-fusion stage and a reliance on only the SVM classifier rather than other neural network and deep learning methods.

Combining the benefits of LSTM and CNN, a deep-learning architecture is proposed by Dar et al.^6^, which has proved to be effective at recognizing emotions and outperforming previous approaches. Their method is subject-agnostic, combining two publicly available data sets (DREAMER and AMIGOS) with low-cost, wearable sensors to extract physiological signals suitable for real-world situations. Multimodal fusion achieves the maximum overall accuracy for the AMIGOS and DREAMER datasets. However, the scope of the research is limited to the decision-level fusing of modalities using majority voting.

The GCB-Net was used to recognize emotions using EEG-channel inputs by Zhang et al.^7^. They use a graph convolutional layer to deal with the network-structured input before stacking numerous standard CNN layers to abstract high-level features. They used two datasets called DREAMER and SEED to compare the efficiency of the proposed GCB-Net. GCB-Net outperformed other models with identical settings in the DREAMER dataset. The experiment also discovered that the GCB-Net and BLS had strong classification abilities in EEG emotion recognition. However, the extraction of additional relevant features is lacking in this study.

To facilitate more discriminative EEG feature extraction, a novel Dynamical Graph Convolutional Neural Network (DGCNN)-based multichannel EEG emotion recognition algorithm was proposed by Song et al.^8^. The proposed DGCNN method employs a neural network to dynamically learn the underlying relationship between multiple electroencephalogram (EEG) channels, which is represented by an adjacency matrix. The SJTU emotion EEG datasets, SEED and DREAMER, are heavily used in their research. The results demonstrated that the DGCNN methodology beats state-of-the-art approaches in terms of recognition performance. In terms of average valence, the suggested DGCNN beats SVM, Graph SLDA, and GSCCA on the DREAMER dataset.

Zheng et al.^9^ used several classifiers, including K-Nearest neighbor, Support-vector machine, Graph regularized extreme learning machine, DBN, and DBN-HMM in their proposed approach for analyzing EEG signals and comparing the performance with other deep learning models. The experimental results showed that the high-frequency (beta and gamma) characteristics were associated with emotional recognition more closely, and DBN and DBN-HMM obtained the most reliable results.

Using a dataset from the International Affective Picture System, Bhardwaj et al.^10^ extracted features from EEG signals by using Power Spectral Density and Energy. Data pre-processing was done by using segmentation, Band-pass filtering, and Independent Component Analysis before features were extracted (ICA). Finally, EEG signals were classified into seven different emotions using machine learning approaches including Linear Discriminant Analysis (LDA) and Support Vector Machine (SVM).

Human emotion is classified using EEG signals in research^4^, where audiovisual stimulation dependent protocols were designed to obtain EEG signals using 63 sensors. Using a neural network, the authors studied the EEG signals by means of a Discrete Transformation and grouped them into five frequency sub-bands using the ‘db4’ Wavelet Transformation, while two statistical characteristics were derived from the alpha band. The study showed that the audio visual feedback performed better than the visual stimulation.

Another study^11^ compared auditory and visual elicitors for emotional prediction. The data was obtained from 24 participants, including facial electromyography, electroencephalography, skin conductivity, and respiratory data, from various physiological signals for six selected emotions. The authors employed two methods of data mining which are decision rules and K-nearest neighbor and extracted the features from physiological measurements. When it came to prediction accuracy, there were no clear cultural differences between Chinese and Indian participants. These findings suggested to the authors that auditory stimuli can be better understood as emotional elicitors, which could have implications for HCI applications.

A novel approach for understanding emotions from EEG signals is suggested by Paul et al.^12^. Eight stable participants were used for data collection, and the EEG signals were collected by seven EEG amplifier channels. Multi-fractal Detrended Fluctuation Analysis (MFDFA) approach is used to extract the features from each channel for the entire frequency bands. Authors implemented some efficient classifiers including Support Vector Machine, Linear Discriminant Analysis, Quadratic Discriminant Analysis, and K Nearest Neighbor.

Empirical mode of decomposition is used in research^13^ for emotional identification using EEG signals. The dataset of the channelled BIOPAC laboratory device was obtained and EEG signals were gained from a capacity of 13 women and 13 men for 12 nice and 12 frustrating images. Empirical Mode Decomposition (EMD) has been used to test nonlinear and also the non-stationary time series that have interrupted the signal into IMFs. The authors used different Classifiers—SVM, LDA, Naive Bayes to classify pleasant and unpleasant emotions and compared their performances. In short, the authors found that SVM provides a better solution by using EMD methods from EEG signals.

The emotion detection of EEG brain signals using the Support Vector Machine (SVM) is discussed in Ref.^14^ and the authors have used emotional stimuli from the International Affective Picture System. The study recognized five kinds of feelings, such as happy, relaxed, optimistic, sad, and frightened. Implemented a feature extraction process. Via SVM, the combined feature set of all subjects was processed. The findings showed that 70% correct in emotion detection in the arousal-valence domain among more than 30 samples.

Different machine learning algorithms were applied to describe the emotion as a psychological measure in research^15^. The authors suggested an approach to describe the various kinds of estimations with better precision in humans. The authors extracted several features from EEG brain signals and used the correlation matrix, the data benefit assessment and the recursive process of eliminating features to refine the collection of features. Then the Extreme Gradient Boosting algorithm (XGBoost) was used as the classification technique. The DEAP data set was used as a checked method, and numerous classification algorithms including Naive Bayes, KNN, C4.5, Decision Tree, Random Forest algorithms were used in relation to the proposed solution. The DEAP algorithm is based on the structure of gradient boosting and follows linear model solver and tree learning.

**Table 1:** Comparison with state-of-the-art related work on DREAMER dataset. A, V and D represent arousal, valence and dominance respectively.

Study	Methodology	Target	Accuracy
Katsigiannis et al.^3^	Statistical features, SVM	High/low arousal High/low valence High/low dominance	62.17% (A) 62.49% (V) 61.84% (D)
Dar et al.^6^	LSTM + CNN	High valence-high arousal High valence-low arousal Low valence-high arousal Low valence-low arousal	90.8%
Tuncer et al.^5^	Textural, statistical and fused features, SVM	High/low arousal High/low valence High/low dominance	94.58% (A) 94.44% (V) 92.86% (D)
Zhang et al.^7^	GCB-Net	High/low arousal High/low valence High/low dominance	89.32% (A) 86.99% (V) 89.20% (D)
Song et al.^8^	DGCNN	High/low arousal High/low valence High/low dominance	84.54% (A) 86.23% (V) 85.02% (D)
Proposed method	Spectrogram features, 2DCNN + XGBoost	High/low arousal High/low valence High/low dominance	99.712% (A) 99.770% (V) 99.770% (D)

In summary, in earlier studies, statistical features, frequency domain features, and time domain features have been extracted, which needed additional domain expertise and the requirement to understand the impact of a particular feature for classifying arousal, valence, and dominance. However, in our proposed method, we directly generate spectrogram images from the raw EEG signals and extract both the shallow and depth-level image features for affective state classification. We observed that the spectrogram image contains more discriminative properties for classifying arousal, valence, and dominance.

## Proposed method

Figure 1, illustrates the proposed method, which is generally divided into two segments. On the left, we take a feature fusion-based approach, emphasizing signal processing on the acquired dataset by denoising it with a band pass filter and extracting alpha, beta, and theta bands for further processing. Numerous features have been extracted from the extracted bands. Feature extraction methods include the Fast Fourier Transform, Discrete Cosine Transform, Poincare, Power Spectral Density, Hjorth parameters, and some statistical features. The Chi-square and Recursive Feature Elimination procedures were used to choose the discriminative features among them. Finally, we utilized classification methods such as Support Vector Machine and Extreme Gradient Boosting to classify all the dimensions of emotion and obtain accuracy scores. On the other hand, we take a spectrogram image-based 2DCNN-XGBoost fusion approach, where we utilize a bandpass filter to denoise the data in the region of interest for different cognitive states. Following that, we performed the Short-Time Fourier Transform and obtained spectrogram images. To train the model on the retrieved images, we use a two-dimensional Convolutional Neural Network (CNN) and a dense layer of neural network to obtain the retrieved features from CNN’s trained layer. After that, we utilized Extreme Gradient Boosting to classify all of the dimensions of emotion based on the retrieved features. Finally, we compared the outcomes from both approaches.

### Data description

In the proposed method (i.e., Fig. 1), we have used the DREAMER^3^ dataset. Audio and video stimuli were used to develop the emotional responses of the participants in this dataset. This dataset consists of 18 stimuli tested on participants, and Gabert-Quillen et al.^16^ selected and analyzed them to induce emotional sensation. The clips came from several films showing a wide variety of feelings. Two of each film centered on one emotion: amusement, excitement, happiness, calm, anger, disgust, fear, sad, and surprise. All of the clips are between 65 and 393 seconds long, giving users plenty of time to convey their feelings^17,18^. However, just the last 60 s of the video recordings were considered for the next steps of the study. The clips were shown to the participants on a 45-inch television monitor with an attached speaker so that they could hear the soundtrack and put them to the test. The EEG signals were captured with the EMOTIV EPOC, a 16-channel wireless headset. Data from sixteen distinct places were acquired using these channels. The wireless SHIMMER ECG sensor provided additional data. This study, however, focused solely on EEG signals from the DREAMER dataset.

Initially, the data collection was performed for 25 participants, but due to some technical problems, data collection from 2 of them was incomplete. As a result, the data from 23 participants were included in the final dataset. The dataset consists of signals from trail and pre-trail. Both were collected as a baseline for each stimuli test. The data dimension of EEG signals from the DREAMER dataset is shown in Table 2.

### Signal pre-processing

EEG signals usually have a lot of noise in them. As a result, the great majority of ocular artifacts occur below 4 Hz, muscular motions occur above 30 Hz, and power line noise occurs between 50 and 60 Hz^3^. For a better analysis, the noise must be decreased or eliminated. Additionally, to work on a specific area, we must concentrate on the frequency range that provides us with the stimuli-induced signals. The information linked to the emotion recognition task is included in a frequency band ranging from 4 to 30 Hz^3^. We utilized band pass filtering to acquire sample values ranging from 4-30 Hz to remove the noise from the signals and discover the band of interest.

#### Band pass filter

The band-pass filter is a technique or procedure that accepts frequencies within a specified range of frequency bands while rejecting any frequencies above the frequency of interest. The bandpass filter is a technique that uses a combination of a low pass and a high pass filter to eliminate frequencies that aren’t required. The fundamental goal of such a filter is to limit the signal’s bandwidth, allowing us to acquire the signal we need from the frequency range we require while also reducing unwanted noise by blocking frequency regions we won’t be using anyway. In both sections of our proposed method, we used a band-pass filter. On the feature fusion-based approach, we used this filtering technique to filter the frequency band between 4 and 30 Hz, which contains the crucial information we require. This helps in the elimination of unwanted noises. We’ve decided to divide the signals of interest into three more bands: theta, alpha, and beta. These bands were chosen because they are the most commonly used bands for EEG signal analysis. The defining of band borders is somewhat subjective. The ranges that we use in our case are theta ranging between 4 and 8Hz, alpha ranging between 8 and 13 Hz, and beta ranging between 13 and 20 Hz. For the 2DCNN-XGBoost fusion-based approach, using this filter technique, we filtered the frequency range between 4 and 30 Hz, which contains relevant signals and generated spectrum images. Here the spectrograms from the signals were extracted using STFT and transformed into RGB pictures.

**Table 2:** Data dimension of EEG signals from the DREAMER dataset.

Attribute	Amount/details
Number of participants	25 (23)
Stimuli amount	18
Video contents	Audio, video
Duration of contents	65–393 s
Age of participants	22–33
Signals	EEG
Channels	14
Sampling rate	128 Hz
Rating of	Valence, arousal and dominance
Range of rating	0–5
Total data	(23 $\times$ 18) = 414
Spectrogram images	(23 $\times$ 18 $\times$ 14) = 5796

### Feature extraction

After pre-processing, we have used several feature extraction techniques for our feature fusion-based and the 2DCNN-XGBoost fusion-based approach that we discussed below:

#### Fast Fourier transform

Fast Fourier transform is among the most useful methods for processing various signals^19–23^. We used the FFT algorithm to calculate a sequence of Discrete Fourier Transform. The FFT stems are evaluated because they operate in the frequency domain, in the time or space, equally computer-feasible. The O(NlogN) result can also be determined by the FFT. Where N is the length of the vector. It functions by splitting a N time-domain signal into a N time domain with one single stage. The second stage is an estimate of the N frequency range for the N time-domain signals. Lastly, the N spectrum was synthesized into one frequency continuum to speed up the Convolutional Neural Network training phase.

The equations of FFT are shown below (1), (2):

1 $$\begin{aligned} H(p)= & {} \sum _{t=0}^{N-1} r(t) W_{N}^{p n}, \end{aligned}$$

2 $$\begin{aligned} r(t)= & {} \frac{1}{N} \sum _{p=0}^{N-1} H(p) W_{N}^{-p n}. \end{aligned}$$

Here $H_p$ represents the Fourier co efficients of r(t)

**Figure 2:**
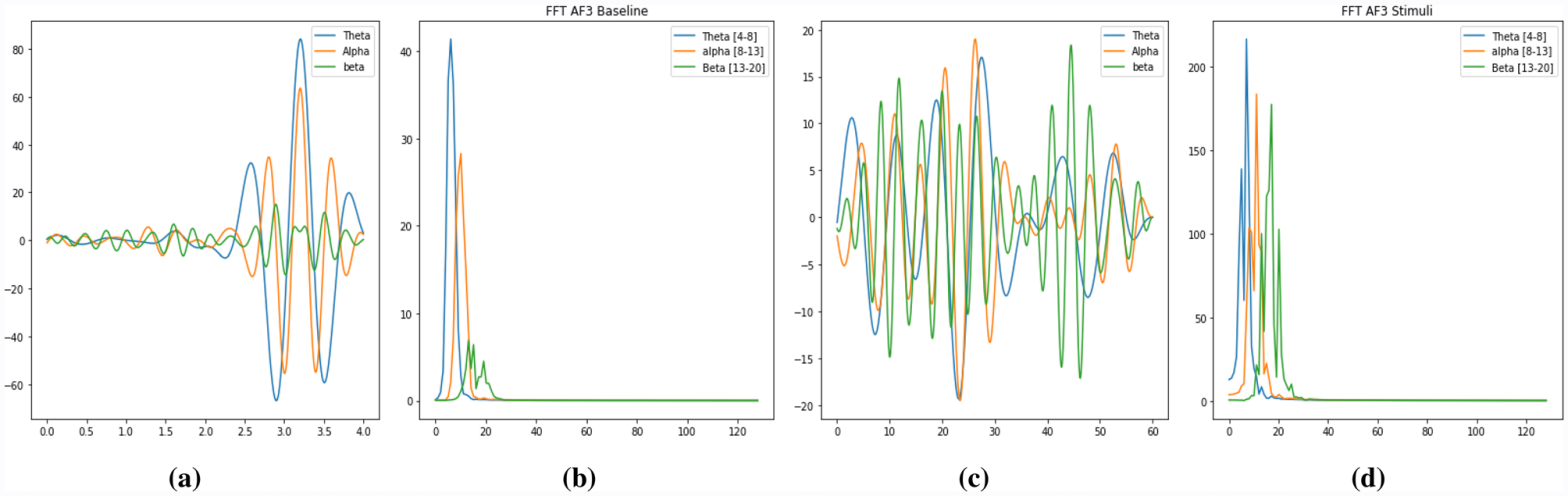
(**a**) A baseline EEG signal in time domain, (**b**) A baseline EEG signal in frequency domain using FFT, (**c**) A stimuli EEG signal in time domain, (**d**) A stimuli EEG signal in frequency domain using FFT .

We have implemented this FFT to get the coefficients shown in Fig. 2. The mean and maximum features for each band were then computed. Therefore, we get 6 features for each channel across 3 bands, for a total of 84 features distributed across 14 channels.

#### Discrete cosine transformation

This method exhibits a finite set of data points for all cosine functions at varying frequencies which is used in research^24–28^. The Discrete Cosine Transformation (DCT) is usually applied to the coefficients of a periodically and symmetrically extended sequence in the Fourier Series. In signal processing, DCT is the most commonly used transformation method (TDAC).The imaginary part of the signal is zero in the time domain and in the frequency domain. The actual part of the spectrum is symmetrical, the imaginary part is unusual. With the following Eq. (3) , we can transform normal frequencies to the mel frequency:

3 $$\begin{aligned} X_{P}=\sum _{n=0}^{N-1} x_{n} \cos {\left[ \frac{\pi }{N}\left( n+\frac{1}{2}\right) P\right] }, \end{aligned}$$

where, N is the the list of real numbers and $X_p$ is the set of N data values

**Figure 3:**
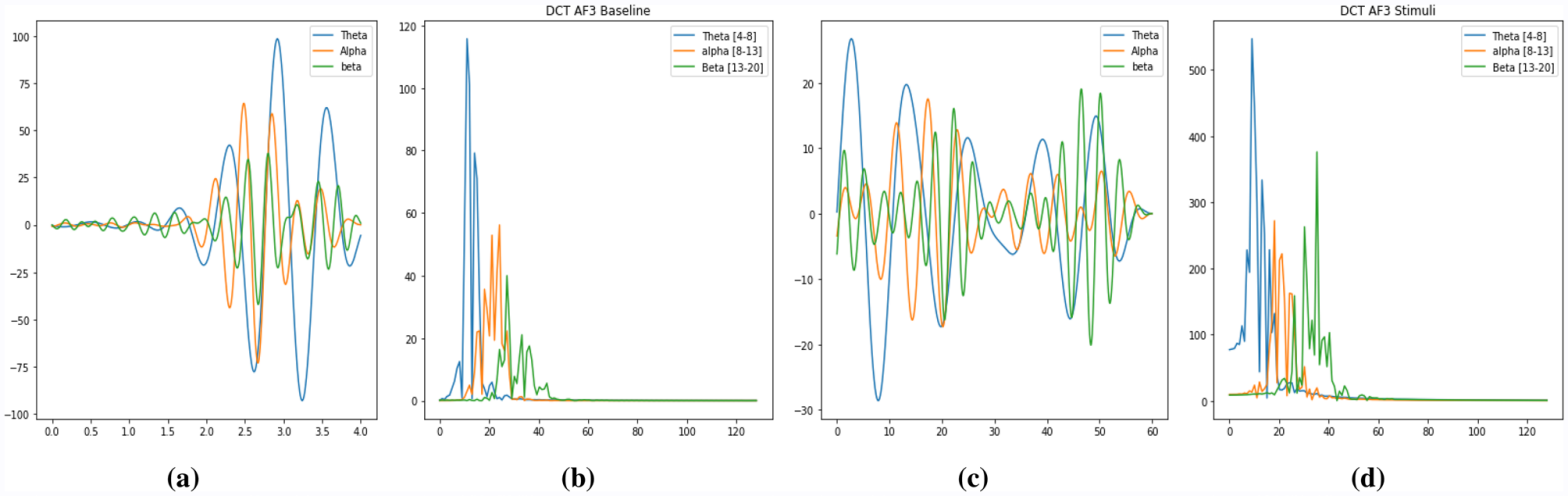
(**a**) A baseline EEG signal in time domain, (**b**) A baseline EEG signal in frequency domain using DCT, (**c**) A stimuli EEG signal in time domain, (**d**) A stimuli EEG signal in frequency domain using DCT.

We have implemented DCT to get the coefficients shown in Fig. 3. The mean and maximum features for each band were then computed. Therefore, we get 6 features for each channel across 3 bands, for a total of 84 features distributed across 14 channels.

#### Hjorth parameters

The Hjorth parameter is one of the ways in which a signal’s statistical property is indicated in the time domain and has three parameters which are Activity, Mobility, and Complexity. These parameters were calculated in many research^29–32^.

Activity: The parameter describes the power of the signal and, the variance of a time function. This can suggest the power spectrum surface within the frequency domain. The notation for activity is given below (4),

4 $$\begin{aligned} var(y(t)). \end{aligned}$$

Mobility: The parameter represents the average frequency or the share of the natural variation of the spectrum. This is defined as the square root of the variance of the first y(t) signal derivative, which is divided by the y(t). The notation for activity is given below (5),

5 $$\begin{aligned} \sqrt{\frac{var(y'(t))}{var(y(t))}}. \end{aligned}$$

Complexity: The parameter reflects the frequency shift. The parameter contrasts the signal resemblance with a pure sinusoidal wave, where the value converges to 1 if the signal is more identical. The notation for activity is given below (6),

6 $$\begin{aligned} \frac{mobility(y'(t))}{mobility(y(t))}. \end{aligned}$$

For our analysis, we calculated Hjorth’s activity, mobility, and complexity parameters as features. Therefore, we get 9 features for each channel across 3 bands, for a total of 126 features distributed across 14 channels.

#### Statistical features

Statistics is the application of applied or scientific data processing using mathematics. We use statistical features to work on information-based data, focusing on the mathematical results of this information. We can learn and gain more and more detailed information on how statistics arrange our data in particular and how other data science methods can be optimally used to achieve more accurate and structural solutions. There is multiple research^33–35^ on emotion analysis where statistical features were used. The statistical features that we have extracted are median, mean, max, skewness and variance. As a result, we get 5 features for each channel, for a total of 70 features distributed across 14 channels.

#### Poincare

The Poincare, which takes a series of intervals and plots each interval against the following interval, is an emerging analysis technique. In clinical settings, the geometry of this plot has been shown to differentiate between healthy and unhealthy subjects. It is also used in a time series for visualizing and quantifying the association between two consecutive data points. Since long-term correlation and memory are demonstrated in the dynamics of variations in physiological rhythms, this analysis was meant to expand the plot of Poincare by steps, instead of between two consecutive points, the association between sequential data points in a time sequence. We used two parameters in our paper which are:

SD1: Represent standard deviation from axis 1 of the distances of points and defines the width from the ellipse (short-term variability). Descriptors SD1 can be defined as (7):

7 $$\begin{aligned} SD1 = \frac{\sqrt{2}}{2}SD(P_n - P_{n+1}). \end{aligned}$$

SD2: The standard deviations from axis 2 and ellipse length (long-term variability) are equivalent to SD2.Descriptors SD2 can be defined as (8):

8 $$\begin{aligned} SD2 = \sqrt{2SD(P_n)^2 - \frac{1}{2}SD(P_n - P_{n+1})^2}. \end{aligned}$$

We have extracted 2 features which are SD1 and SD2 from each band (theta, alpha, beta). Therefore, we get 6 features for each channel across 3 bands, for a total of 84 features distributed across 14 channels.

#### Power spectral density

The Welch method is a modified segmentation system and is used to assess the average periodogram, which is used in papers^3,23,36^. The Welch method is applied to a time series. For spectral density, it is concerned with decreasing the variance in the results. Power Spectral Density (PSD) informs us which differences in frequency ranges are high and could be very helpful for further study.The Welch method of the PSD can usually be described by the following equations: (9), (10) of the power spectra.

9 $$\begin{aligned} P(f)= & {} \frac{1}{M U}\left| \sum _{n=0}^{M-1} x_{i}(n) w(n) e^{-j 2 \pi f}\right| ^{2}, \end{aligned}$$

10 $$\begin{aligned} P_{\text{ welch } }(f)= & {} \frac{1}{L} \sum _{i=0}^{L-1} P(f). \end{aligned}$$

Here, the equation of density is defined first. Then, Welch Power Spectrum implies that for each interval, the average time is expressed. We have implemented this Welch method to get the PSD of the signal. From that, the mean power has been extracted from each band. As a result, we get 3 features for each channel across 3 bands, for a total of 42 features distributed across 14 channels.

#### Short-Time Fourier Transform

**Figure 4:**
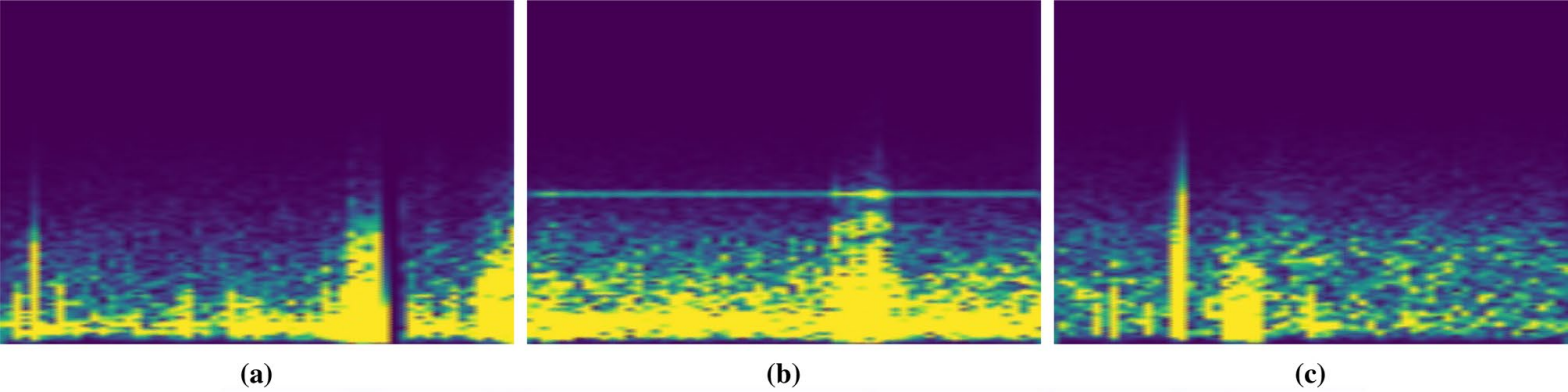
EEG signal spectrograms using STFT with classification (**a**) high arousal, high valence, and low dominance, (**b**) low arousal, high valence, and high dominance, (**c**) high arousal, low valence, and low dominance.

A Convolutional Neural Network (CNN) is primarily used to process images since the time series is converted into a time-frequency diagram using a Short-Time Fourier Transform (STFT). It extracts required information from input images using multilayer convolution and pooling, and then classifies the image using fully connected layers. We have calculated the STFT using the filtered signal, which ranges between 4 and 30 Hz, and transformed them into RGB images. Some of the generated images are shown in Fig. 4.

To convert time series EEG signals into picture representations, Wavelet algorithms and Fourier Transforms are commonly utilized, which we have used in our secondary process. But in order to preserve the integrity of the original data, EEG conversion should be done solely in the time-frequency domain. As a result, STFT is the best method for preserving the EEG signal’s most complete anesthetic characteristics, which we have used in our second process. The spectrograms from the signal were extracted using STFT and the Eq. (11) is given below:

11 $$\begin{aligned} Z_{n}^ {e^{(j \hat{\omega )}}}=e^{-j\hat{\omega }n}[(W(n)e^{j\hat{\omega }n}) \times x(n)], \end{aligned}$$

where, $e^{-j\hat{\omega }n}$ is the complex bandpass filter output modulated by signal. From the above equation we have calculated the STFT from the filtered signals.

### Feature ratio

For our feature fusion-based approach, as we have pre-trail signals, we have used 4 s of pre-trail signals as baseline signals, resulting in 512 samples for each at a 128 Hz sampling rate. Then similar to the features extracted for stimuli, the features from baseline signals were also extracted. Then the stimuli features were divided by the baseline features, in order to get only the differences which can be noticed for the feature fusion-based approach by the stimuli test only, which is also done in the paper^3^.

### Feature scaling

After extracting all the features and calculating the ratio between stimuli features and baseline features, we have added the self-assessment ratings of arousal valence and dominance. Now the data set for the feature fusion-based approach has 414 data points with 630 features for each data point. We scaled the data using Min–Max Scaling to remove the large variation in our data set. The estimator in Min–Max Scaling scales and translates each value individually so that it is between 0 and 1, within the defined range.

The formula for Min–Max scale is (12),

12 $$\begin{aligned} X_{n e w}=\frac{X_{i}-{\text {Min}}(X)}{{\text {Max}}(X)-{\text {Min}}(X)}. \end{aligned}$$

### Feature selection techniques

**Figure 5:**
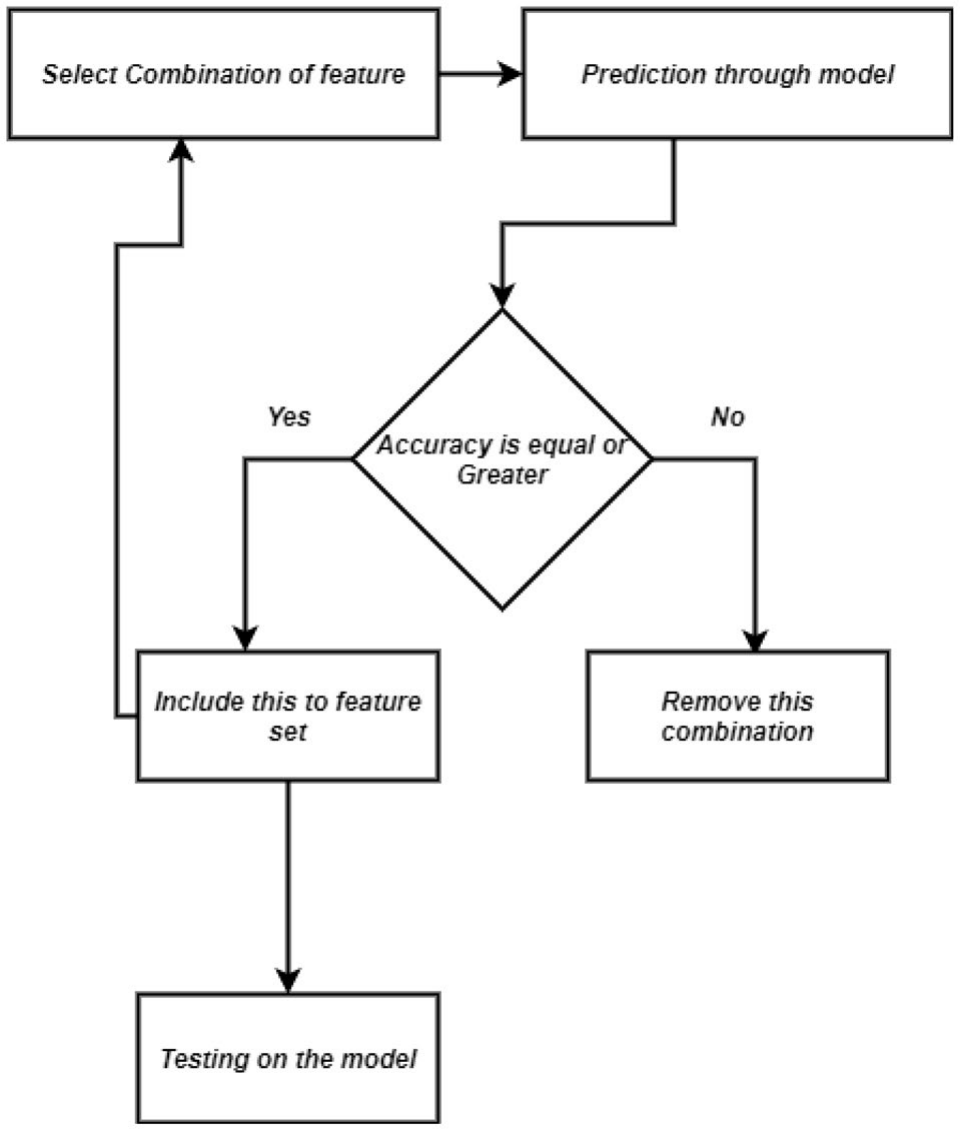
Procedure of recursive feature elimination (RFE).

**Figure 6:**
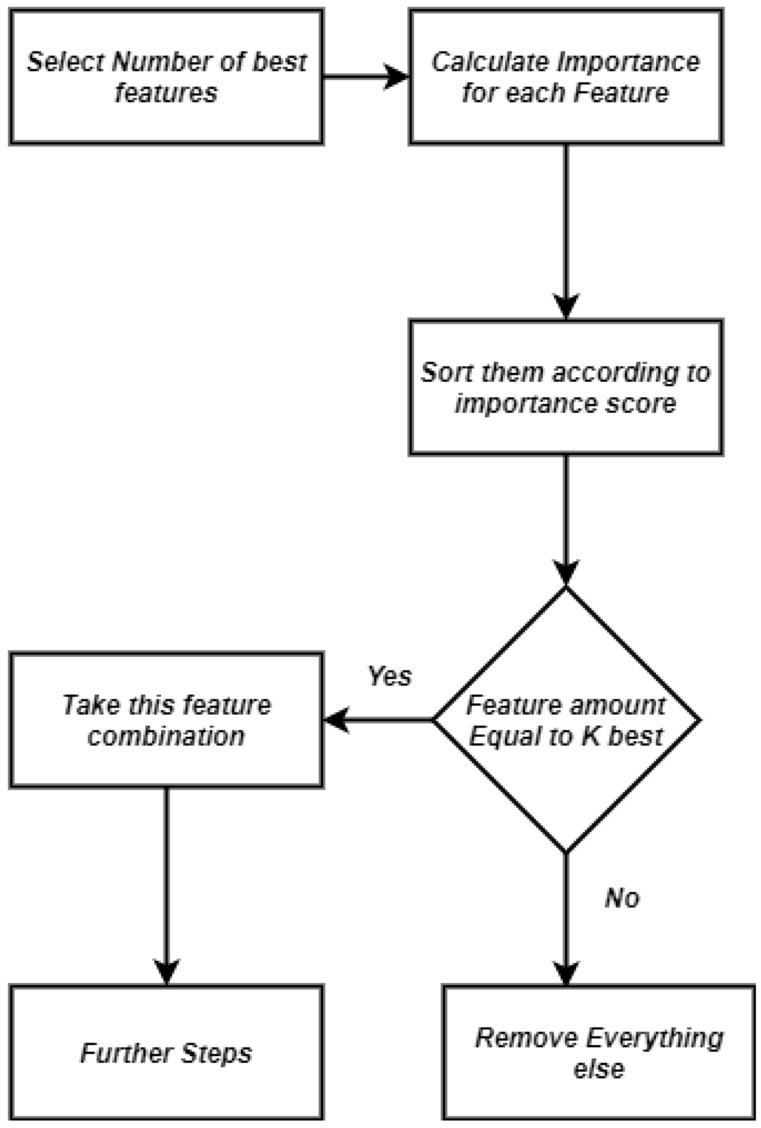
Procedure of feature selection using Chi-square.

There are various feature selection techniques which are used by many researchers, to reduce the number of features which are not needed and only the important features which can play a big role in the prediction. So in our paper we used two feature elimination methods. One is Recursive Feature Elimination (i.e., Fig. 5) and another one is Chi-square test (i.e., Fig. 6) .

#### Recursive feature elimination

RFE (i.e., Fig. 5) is a wrapper type feature selection technique amongst the vast span of features. Here the term recursive is representative of the loop work of this method that traverses backward on loops to identify the best fitted feature giving each predictor an importance score and later eliminating the least importance scored predictor. Additionally cross-validation is used to find the optimal number of features to rank various feature subcategories and pick the best selection of features for scoring. In this method one attribute is taken and along with the target attribute and this procedure keeps forwarding combining attributes and merging with the target attribute to produce a new model. Thus different subsets of features of different combinations generate models through training. All these models are then strained out to get the maximum accuracy resulting model and its consecutive features. In short, we remove those features which result in the accuracy to be high or at least equal and return it back if the accuracy gets low after elimination . Here we have used step size of 1 to eliminate one feature at a time at each level which can help to remove the worst features early, keeping the best features in order to improve the already calculated accuracy of the overall model.

#### Chi-square

Chi-square (i.e., Fig. 6) test is a filter method that states the accuracy of a system comparing the predicted data with the observed data based on their importance. It is a test that figures out if there is any feature effective on nominal categorized data or not in order to compare between observed and expected data. In this method one predicted data set is considered as a base point and expected data is calculated from the observed value with respect to the base point.

The Chi-square value is computed by (13):

13 $$\begin{aligned} \chi ^{2}=\sum _{i=1}^{m} \sum _{j=1}^{k} \frac{\left( A_{i j}-\frac{R_{i} \cdot C_{j}}{N}\right) ^{2}}{\frac{R_{i} \cdot C_{j}}{N}}, \end{aligned}$$

where, m is the number of intervals, k is the amount of classes, $R_i$ is the amount of patterns in the i range, $C_j$ is the amount of patterns in the j range, $A_{ij}$ is the amount of patterns in i and j range.

After applying RFE and Chi-square , from the achieved accuracy we have observed that, Chi-square does not incorporate a machine learning (ML) model, while RFE uses a machine learning model and trains it to decide whether it is relevant or not. Moreover, in our research, Chi-square methods failed to choose the best subset of features which can provide better results,but because of the extensive nature, RFE methods give the best subset of features mostly in our research. Therefore we consider RFE over Chi-square for feature elimination.

### Class distribution of self assessment ratings

In research^3^, on this data set, they have calculated the mean and standard deviation for the self assessment. Then they have divided each dimension into two classes, high or low. The boundary between high and low was in the mid point of (0-*5) which is 2.5. But we have adjusted this boundary on our secondary process based on some of our observation. We have also calculated the mean and standard deviation of self assessment ratings, shown in Table 3, to separate each dimension of emotions into two separate classes, which will be high (1) and low (0) and will be representing two emotional category for each dimension.

**Table 3:** Mean and standard deviation of self assessment ratings of arousal, valence and dominance.

Stimuli	Concentrated	Mean ± SD	Mean ± SD	Mean ± SD
Number	Emotion	Valence	Arousal	Dominance
1	Calm	3.17 ± 0.72	2.26 ± 0.75	2.09 ± 0.73
2	Surprising	3.04 ± 0.88	3.00 ± 1.00	2.70 ± 0.88
3	Amuse	4.57 ± 0.73	3.83 ± 0.83	3.83 ± 0.72
4	Fear	2.04 ± 1.02	4.26 ± 0.69	4.13 ± 0.87
5	Excite	3.22 ± 1.17	3.70 ± 0.70	3.52 ± 0.95
6	Disgusting	2.70 ± 1.55	3.83 ± 0.83	4.04 ± 0.98
7	Happy	4.52 ± 0.59	3.17 ± 0.98	3.57 ± 0.99
8	Angry	1.35 ± 0.65	3.96 ± 0.77	4.35 ± 0.65
9	Sad	1.39 ± 0.66	3.00 ± 1.09	3.48 ± 0.95
10	Disgusting	2.17 ± 1.15	3.30 ± 1.02	3.61 ± 0.89
11	Calm	3.96 ± 0.64	1.96 ± 0.82	2.61 ± 0.89
12	Amuse	3.96 ± 0.56	2.61 ± 0.89	2.70 ± 0.82
13	Happy	4.39 ± 0.66	3.70 ± 0.97	3.74 ± 0.96
14	Angry	2.35 ± 0.65	2.22 ± 0.85	2.39 ± 0.72
15	Fear	2.48 ± 0.85	3.09 ± 1.00	3.22 ± 0.9
16	Excite	3.65 ± 0.65	3.35 ± 1.07	3.26 ± 1.14
17	Sad	1.52 ± 0.59	3.00 ± 0.74	3.96 ± 0.77
18	Surprising	2.65 ± 0.78	3.91 ± 0.85	3.57 ± 1.04

Arousal: For our 2DCNN-XGBoost fusion based approach, (ratings $> 2.5$) is considered in the class of Excited/Alert and (ratings$< 2.5$) is considered as Uninterested/Bored (0). Here, from the 5796 data, 4200 was in the excited/alert class (1) and 1596 was in the uninterested/bored class. For the feature fusion-based approach, We have focused on the average ratings for excitement which co-responds to stimuli number 5 and 16, having 3.70 ± 0.70 and 3.35 ± 1.07 respectively. Additionally for, calmness, we can take stimuli 1 and 11 into consideration where the average ratings are, 2.26 ± 0.75 and 1.96 ± 0.82 respectively. Therefore, (ratings $> 2$) can be considered in the class of Excited/Alert and (ratings$< 2$) can be considered as Uninterested/Bored. Here, from the 414 data, 393 was in the excited/alert class and 21 was in the uninterested/bored class. We have also shown the parallel Coordinate plot for arousal in Fig. 8a to show the impact of different features on arousal level.

Valence: For our 2DCNN-XGBoost fusion based approach, (ratings $> 2.5$) is considered in the class of happy/elated and (ratings$< 2.5$) is considered as unpleasant/stressed. Here, from the 5796 data, 2254 was in the unpleasant/stressed class and 3542 was in happy/elated class. To store this values in the new data set, unpleasant/stressed is considered as 0 and happy/elated is considered as 1. For the feature fusion-based approach, firstly, we concentrated on the average happiness ratings, which correspond to stimuli 7 and 13, having 4.52 ± 0.59 and 4.39 ± 0.66 respectively. Additionally, stimuli (4, 15) and (6, 10) for fear and disgust were considered where the average ratings are, 2.04 ± 1.02, 2.48 ± 0.85, 2.70 ± 1.55 and 2.17 ± 1.15 respectively. Here, it is clear that, ratings $> 4$ can be considered in the class of happy/elated and ratings$< 4$ can be considered as unpleasant/stressed. Here, from the 414 data, 359 was in the unpleasant/stressed class and 55 was in happy/elated class. To store this values in the new data set, unpleasant/stressed is considered as 0 and happy/elated is considered as 1. We have also shown the parallel Coordinate plot for valence in Fig. 8b to show the impact of different features on valence level.

Dominance: For our 2DCNN-XGBoost fusion based approach, Same approach is followed here with low and high classes. Here, ratings$> 2.5$ in the class of helpless/without control and ratings$< 2.5$ can be considered for the class of empowered. Here, from the 5796 data, 1330 was in the helpless/without control class and 4466 was in empowered class. To store this values in the new data set, helpless/Without Control is considered as 0 and empowered is considered as 1. For the feature fusion-based approach, we have targeted stimuli number 4,6 and 8 which has targeted emotions of fear, disgust and anger, having mean rating of 4.13 ± 0.87, 4.04 ± 0.98 and 4.35 ± 0.65 respectively. So, ratings$> 4$ in the class of helpless/without control and rest for the class of empowered. Here, from the 414 data, 65 was in the helpless/without control class and 349 was in empowered class. To store this values in the new data set, helpless/Without Control is considered as 0 and empowered is considered as 1. We have also shown the parallel Coordinate plot for dominance in Fig. 8c to show the impact of different features on dominance level.

**Figure 7:**
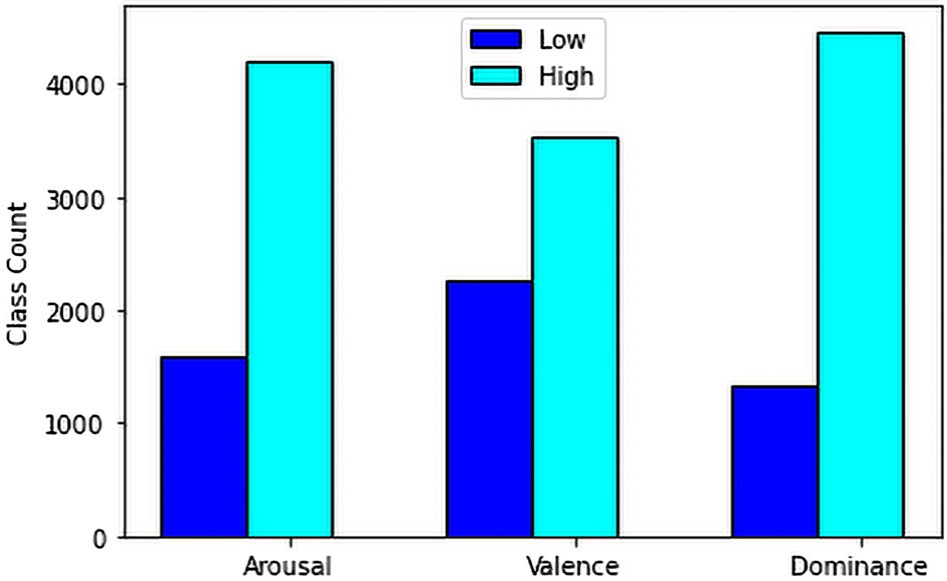
Overall class distribution after conversion to a two-class rating score for arousal, valence and dominance.

The overall class distribution for arousal, valence and dominance is shown in the Fig. 7.

**Figure 8:**
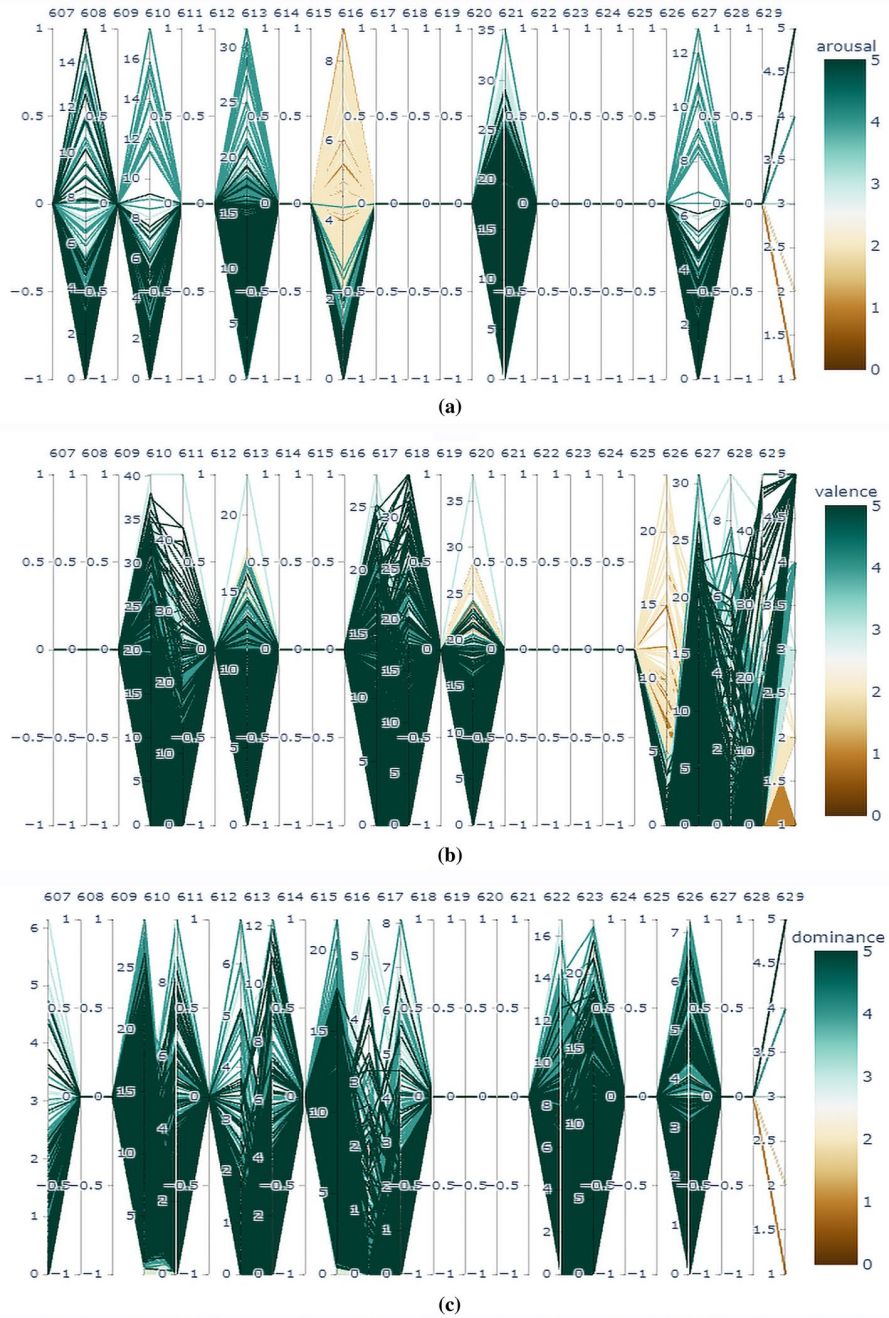
Impact factor of features on (**a**) arousal, (**b**) valence and (**c**) dominance using parallel co-ordinate plot.

### Training through a deep Convolutional Neural Network

**Figure 9:**
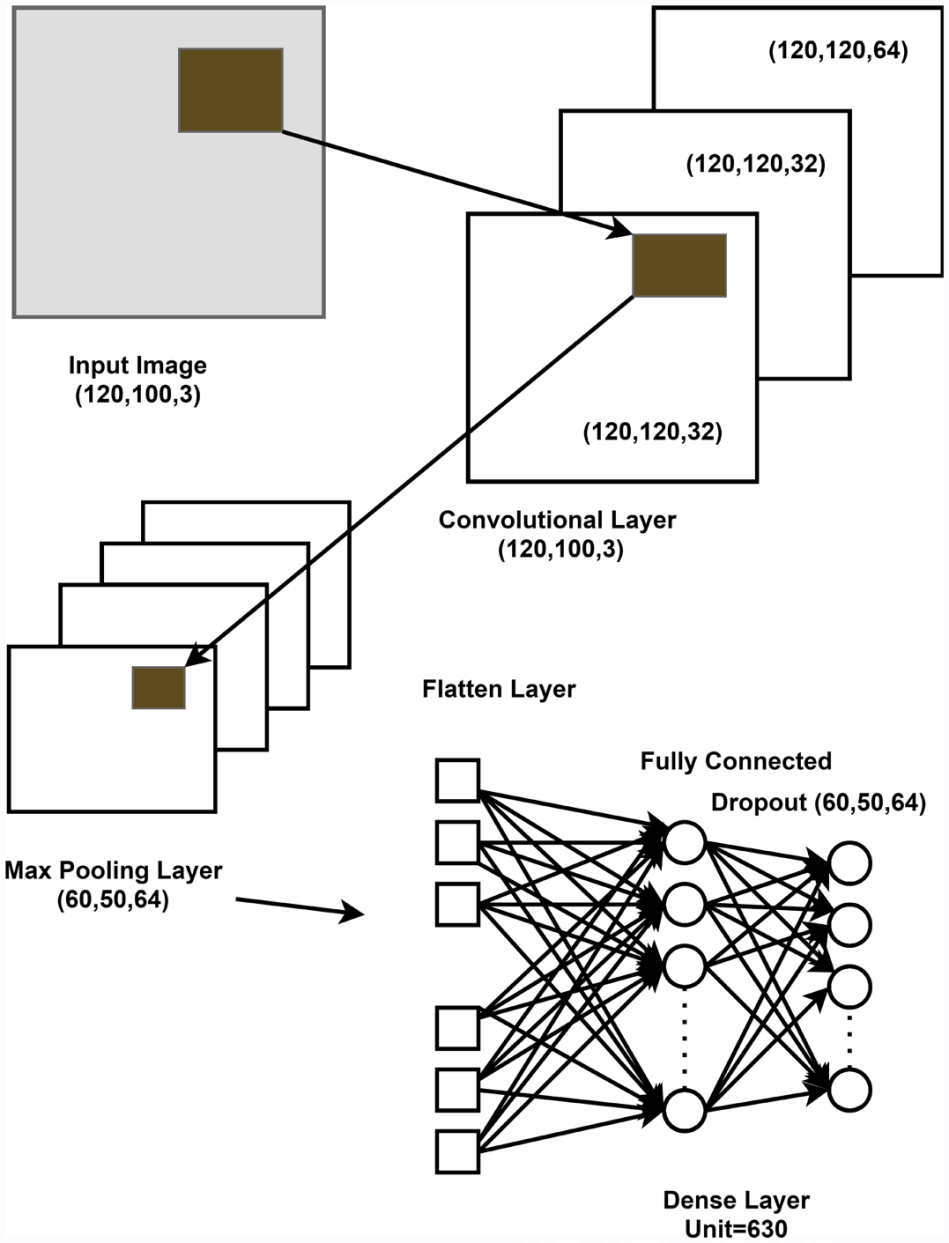
The architecture of the implemented CNN model.

Convolutional Neural Network (CNN) is a type of deep neural network used to analyze visual imagery in deep learning. Figure 9, represents the overall two-dimensional Convolutional Neural Network model used in our proposed method (i.e., Fig. 1), which is also our 2DCNN-XGBoost fusion approach. We generated spectrum images before using this CNN architecture by filtering the frequency band containing significant signals between 4 and 30 Hz. Following that, we compute the Short-Time Fourier Transform of the EEG signals and convert them to spectrogram images before extracting features with a 2D Convolutional Neural Network. We train the model with 2D convolutional layers using the obtained spectrogram images, and then retrieve the trained features from the training layer with the help of another dense layer. We have implemented the test-bed to evaluate the performance of our proposed method. The proposed model is trained using the Convolutional Neural Network (CNN) described below,

#### Conv2D layer

Basic features such as horizontal and diagonal edges are usually extracted by the first layer. This information is passed on to the next layer, which is responsible for detecting more complicated characteristics such as corners and combinational edges. As we progress deeper into the network, it becomes capable of recognizing ever more complex features such as objects, faces, and so on.The classification layer generates a series of confidence ratings (numbers between 0 and 1) on the final convolution layer, indicating how likely the image is to belong to a class. In our proposed method, we have used three layers of Conv2D and identified the classes.

#### Pooling layer

The pooling layer is in charge of shrinking the convolved features spatial size. By lowering the size, the computer power required to process the data is reduced. Pooling can be divided into two types: average pooling and max pooling. We have used max pooling because it gives a better result than average pooling. We found the maximum value of a pixel from a region of the image covered by the kernel using max pooling. It removes all noisy activations and conducts de-noising as well as dimensionality reduction. In general, any pooling function can be represented by the following formula (14):

14 $$\begin{aligned} q_{j}^{(l+1)} = Pool(q_{1}^{(l)}, \ldots ,q_{i}^ {(l)},\ldots ,q_{n}^{(l)}),q_{i}\in R_{j}^{(l)}, \end{aligned}$$

where, $R_{j}^{(l)}$ is the *j*th pooled region at layer l and Pool() is pooling function over the pooled region

#### Dropout layer

We added a dropout layer after the pooling layer to reduce overfitting. The accuracy will continuously improve as the dropout rate decreases, while the loss rate decreases. Some of the max pooling is randomly picked outputs and completely ignored. They aren’t transferred to the following layer.

#### Flatten layer

After a set of 2D convolutions, it’s always necessary to perform a flatten operation.Flattening is the process of turning data into a one-dimensional array for further processing. To make a single lengthy feature vector, we flatten the output of the convolutional layers. It’s also linked to the overall classification scheme.

#### Dense layer

Dense gives the neural network a completely linked layer. All of the preceding layer’s outputs are fed to all of its neurons, with each neuron delivering one output to the following layer.

In our proposed method, with this CNN architecture, diverse kernels are employed in the convolution layer to extract high-level features, resulting in different feature maps. At the end of the CNN model, there is a fully connected layer. The predicted class labels of emotions are generated by the output of the fully connected layer. According to our proposed method, we have added dense layer with 630 units after training layer to extracted this amount of features.

### Extreme Gradient Boosting (XGBoost)

Extreme Gradient Boosting (XGBoost) is a machine learning algorithm that use a supervised learning strategy to accurately predict an objective variable by combining the predictions of several weaker models. It is a common data mining tool with good speed and performance. The XGBoost model computes 10 times faster than the Random Forest model.The XGBoost model was generated utilising the additive tree method, which involves adding a new tree to each step to complement the trees that have already been built.As additional trees are built, the accuracy generally improves. In our proposed model, we have used XGBoost after applying CNN. We extracted some amount of features from CNN’s trained layer. . Then, based on the retrieved features, we used Extreme Gradient Boosting to classify all of the dimensions of emotion. The following Eqs. (15) and (16) are used in Extreme Gradient Boosting.

15 $$\begin{aligned}{}&f(m) \approx f(k)+f^{\prime }(k)(m-a)+\frac{1}{2} f^{n}(k)(m-k)^{2}, \end{aligned}$$

16 $${ \mathcal {L}^{(t)} \simeq \sum _{i=1}^{n}\left[ l\left( q_{i}, q^{(t-1)}\right) +r_{i} f_{t}\left( m_{i}\right) +\frac{1}{2} s_{i} f_{t}^{2}\left( m_{i}\right) \right] +\Omega \left( f_{t}\right) +C },$$

where C is Constant, $r_i$ and $s_i$ are defined as,

17 $$\begin{aligned} r_{i}= & \partial \hat{z}_{i}^{(b-1)}. \int \left( z_{i,} \hat{z}_{i}^{(b-1)}\right) , \end{aligned}$$

18 $$\begin{aligned} s_{i}= & {} \partial \hat{z}_{i}^{(b-1)} .\int \left( z_{i}, \hat{z}_{i}^{(b-1)}\right) . \end{aligned}$$

After removing all the constants, the specific objective at step b becomes,

19 $$\begin{aligned} \sum _{i=1}^{n}\left[ { r_{i}f_{t} }\left( m_{i}\right) +\frac{1}{2}{s_{i} {f}_{t}^{2}(m_{i})}\right] +\Omega \left( f_{t}\right) , \end{aligned}$$

where,

20 $$\begin{aligned} \Omega (f)=\gamma P+\frac{1}{2} \lambda \sum _{j=1}^{P} Z_{j}^{2}, \end{aligned}$$

where $m_i$ is the input, $z_i$ stands for real value known from the training dataset, $\Omega (f)$ is the complexity of the tree, and P is the number of leaves.

## Results and discussion

The DREAMER^3,5–8^, dataset has been used to validate the proposed model. This dataset tracks the emotions induced by audio-visual stimulation through EEG signals. Each participant rated their emotional response on the valence, arousal, and dominance scales after seeing each of the 18 video stimuli intended to represent certain emotions. In addition, participant-specific classification tests for the valence, arousal, and dominance scales were conducted in order to provide baseline findings for the proposed database in terms of classification accuracy and F1 scores. However, because the data set had not been preprocessed, we had to process it ourselves. In the dataset, as there were 23 subjects tested on 18 stimuli with the 14-channel device at a 128 Hz sampling rate, the duration of the recorded signal was 65–393, resulting in an unequal number of sample points. We have just used the final 60 s of the recorded signal in this case. We obtained 7680 points for 60 s of signal at a sample rate of 128 Hz. Furthermore, because there were pre-trail signals in the dataset as a baseline for each stimulus, we only chose 4 s of data from it. So we have 512 pre-trail sample points for 4 s of data at 128 sampling rate. Then we divided each channel into 3 separate bands, which are theta, alpha, and beta. From the extracted band, we have performed feature extraction techniques on both feature fusion and the 2DCNN-XGBoost fusion-based approach.

**Figure 10:**
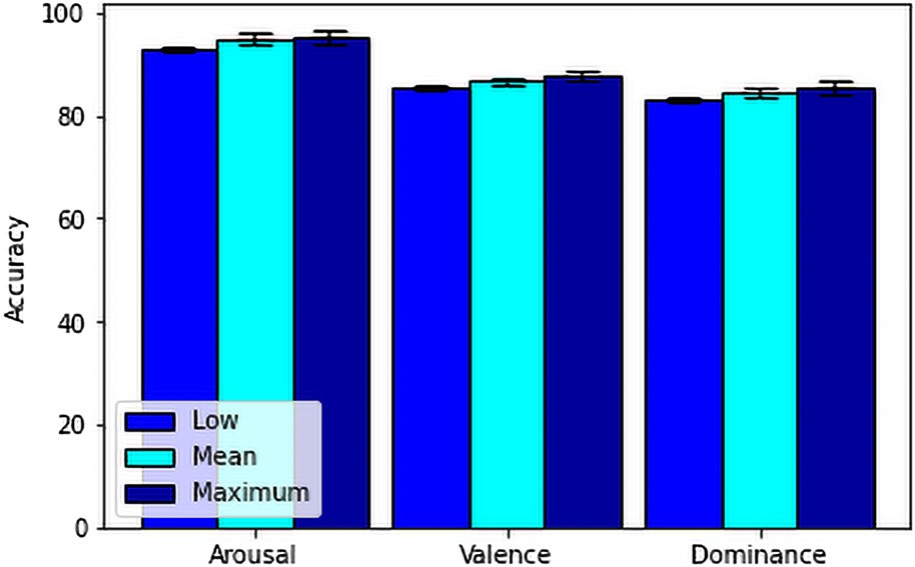
Accuracy comparison between minimum, mean and maximum of the feature fusion-based SVM method.

**Figure 11:**
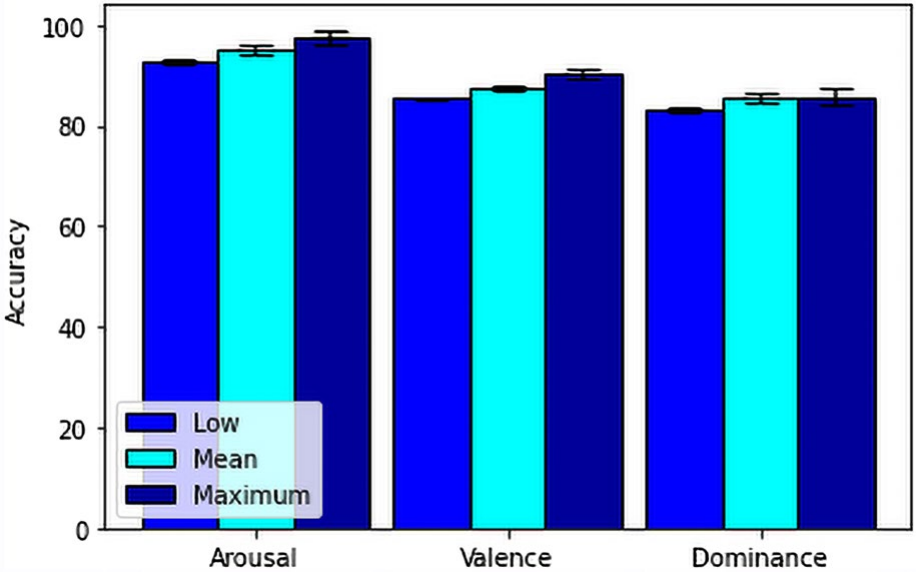
Accuracy comparison between minimum, mean and maximum of the feature fusion-based XGBoost method.

For our feature fusion-based approach, we have used FFT, DCT, Poincare, PSD, Hjorth parameters and some statistical features resulting a total of 630 features from 14 channels for each stimuli test and also for each baseline. After extracting the features, we calculated the ratio between the features from stimuli and baseline for our secondary process only, to get the actual feature values which is affected by the stimuli.Then we have used Chi-square and RFE technique. Here we observed that the RFE technique increased the accuracy of the model.Then we have directly moved to the machine learning algorithms on the final dataset consisting of 414 data from 23 subjects tested on 18 stimuli. For that, firstly, we have used SVM with RBF kernel to test our model on our secondary model.Here, we have also used 10 fold cross validation to calculate the minimum, mean and maximum accuracy of the prediction and showed in Table 4 and Fig. 10. Then we have also used Extreme Gradient Boosting on the final dataset. Here, we have performed 10 fold cross validation for all the dimensions(arousal, valence, dominance) to calculate the minimum, mean and maximum accuracy of the prediction and showed the range and variation between the minimum, mean and maximum in Table 5 and Fig. 11.

**Table 4:** Minimum, mean and maximum accuracy of feature fusion-based SVM method.

Accuracy	Arousal (%)	Valence (%)	Dominance (%)
Minimum	92.857	85.366	82.927
Mean	94.930	86.725	84.309
Maximum	95.238	87.805	85.366

**Table 5:** Minimum, mean and maximum accuracy of feature fusion-based XGBoost method.

Accuracy	Arousal (%)	Valence (%)	Dominance (%)
Minimum	92.857	83.333	82.927
Mean	95.174	87.456	84.541
Maximum	97.561	90.244	85.714

On the other hand, our 2DCNN-XGBoost fusion-based approach from our proposed method performs well on our data set and yields the best results. We have filtered the frequency band between 4 and 30 Hz, which contains significant signals,and generated spectrum images using this filter technique. The spectrograms from the signals were extracted and converted to RGB images using STFT. Here RGB is a virtual image which is a matrix of pixel values with three planes.Each layer creates many activation functions that are passed onto the next layer input an image into a ConvNet such as Conv2D layer, pooling layer, dropout layer, flatten layer, dense layer accordingly. Here we have added dense layer with 630 units after training layer to extracted this amount of features. Finally we have used the features extracted from the trained layer of 2DCNN and used XGBoost algorithm for classification. The results that we have focused on the accuracy score and the AUC score of all three dimensions of emotions which are arousal, valence and dominance. Here AUC is a model where true positive and true negative are better predicted. The overall area beneath the ROC curve is measured by the AUC. The overall accuracy and AUC results are shown in Table 6.

**Table 6:** Accuracy and AUC scores of arousal, valence and dominance of the suggested spectrogram image-based CNN-XGBoost fusion method.

Score	Arousal (%)	Valence (%)	Dominance (%)
Accuracy	99.712	99.770	99.770
AUC	99.607	99.713	99.587

**Figure 12:**
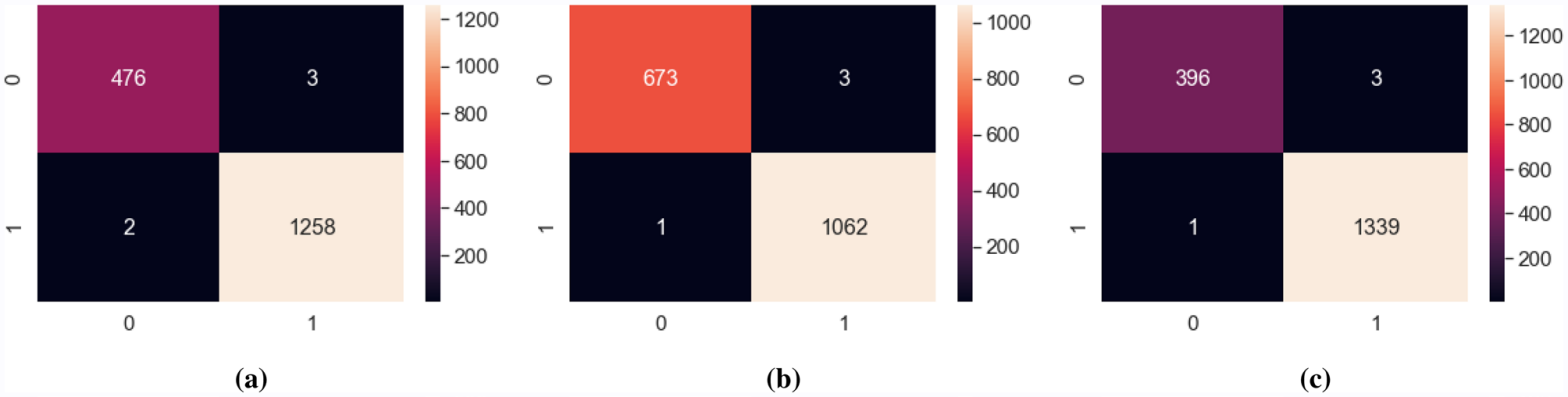
Confusion matrix of (**a**) arousal, (**b**) valence and (**c**) dominance of the proposed spectrogram image based CNN-XGBoost fusion method.

We have also shown the confusion matrix in Fig. 12 for all the dimension of emotion which are arousal, valence and dominance in order to show how much our hybrid model of 2DCNN features and XGboost classification fusion can differentiate between true positive, false positive and true negative and false negative.

**Figure 13:**
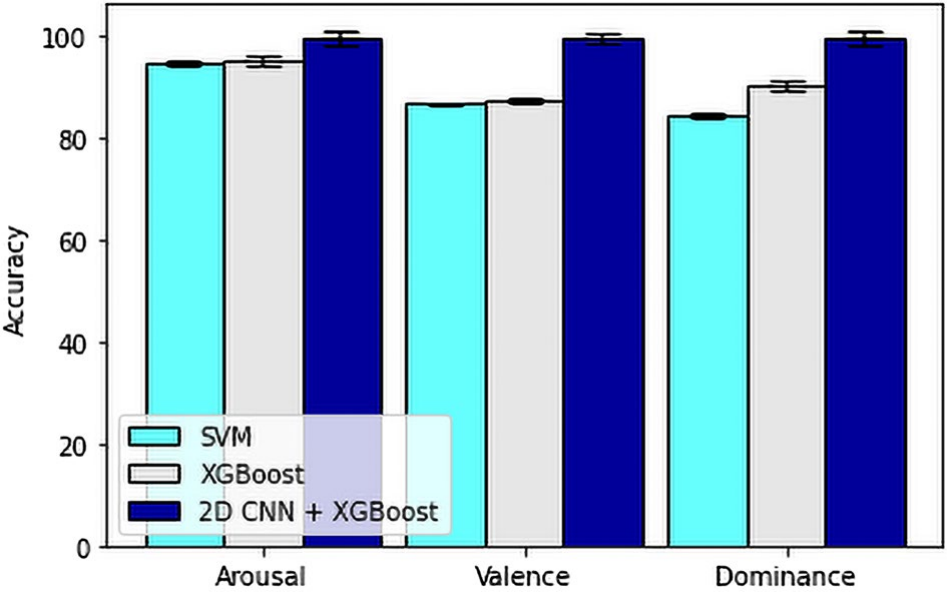
Mean accuracy comparison of arousal, valence and dominance among (i) feature fusion-based SVM method (ii) feature fusion-based XGBoost method, and (iii) the proposed spectrogram image-based CNN-XGBoost fusion method.

For the performance study and overall overview of the results obtained from different approaches, we compared the results of our spectrogram image-based 2DCNN-XGBoost fusion approach and feature fusion-based method of extracting more relevant features and performing classification with Support Vector Machine and XGBoost classification algorithm. In Fig. 13, we have shown the accuracy results in a chart in order to compare between their accuracy and performance. The overall comparison between our proposed method and state-of-the-art related work done on similar dataset are shown in Table 1.

## Conclusion

Human emotional states have been identified as a challenging topic in the domain of affective computing due to their versatility and complexity. However, we used the DREAMER dataset in the proposed method, which comprises EEG signals recorded using a 14-channel device at a 128 Hz sample rate from 23 participants tested on 18 distinct stimuli and their self-assessment ratings. Using this dataset, we present two approaches. The first is a feature fusion-based method with SVM and XGBoost classifiers, and the second is a 2DCNN-XGBoost fusion method based on spectrogram images. The combination of spectrogram image-based 2DCNN features and the Extreme Gradient Boosting classification method provided an accuracy of 99.712% for arousal, 99.770% for valence, and 99.770 % for dominance. This accuracy level is significantly higher than the results we obtained from our feature fusion-based approach with the SVM classifier, which provided a mean accuracy of 94.930% for arousal, 86.725% for valence, and 84.309% for dominance. It is also better than the results we obtained from our feature fusion-based approach with the XGBoost classifier, which provided a mean accuracy of 95.174% for arousal, 87.456% for valence, and 84.541% for dominance. In conclusion, our spectrogram image-based 2DCNN features and classifying with XGBoost provided us with better results, which are significantly higher than the results (i.e., Table 1), found in previous relevant studies done on this data set and our feature fusion-based method. Future dimensions will also help EEG with more specific deep neural networks for greater performance and various psychological signals.

## Data Availability

The DREAMER dataset is available at http://ahcse.uws.ac.uk/index.php?page=projects&project=dreamer.
